# Motives, experiences and psychological strain in medical students engaged in refugee care in a reception center– a mixed-methods approach

**DOI:** 10.1186/s12909-019-1730-8

**Published:** 2019-08-05

**Authors:** David Kindermann, Marie P. Jenne, Carolin Schmid, Kayvan Bozorgmehr, Katharina Wahedi, Florian Junne, Joachim Szecsenyi, Wolfgang Herzog, Christoph Nikendei

**Affiliations:** 1 Department of General Internal Medicine and Psychosomatics, University Hospital Heidelberg, Heidelberg, Germany; 2 Department of General Practice and Health Services Research, University Hospital Heidelberg, Heidelberg, Germany; 3 Department of Psychosomatic Medicine and Psychotherapy, Medical University Hospital Tübingen,University of Tübingen, Tübingen, Germany

**Keywords:** Secondary traumatization, Sense of coherence, Global health electives, Globalization, Cultural awareness

## Abstract

**Background:**

The UN Refugee Agency has reported that an increasing number of people are being forcibly displaced worldwide. Despite this, global health issues, especially initiatives focusing on physical and psychological conditions of refugees, are still rarely considered in medical curricula. Furthermore, there is little evidence regarding the experiences and possible related psychological strain of medical students who work with refugees. Therefore, the present study aimed to investigate motivations, learning achievements and experiences, as well as psychological strain and possible protective factors, in medical students volunteering in a reception center for refugees.

**Methods:**

In this prospective study using a mixed-methods approach, we applied (1) qualitative content analysis of semi-standardized interviews in a pre-post design in a subsample of *n* = 16 students. The aims were to analyze (1a) the students’ motivations and experiences in the reception center, and (1b) the students’ perceived learning achievement. We further administered (2) psychometric questionnaires using a cross-sectional approach to *n* = 62 students in order to examine (2a) the students’ psychological strain, in terms of secondary traumatization, depression, anxiety and health-related quality of life, and (2b) possible protective factors such as attachment style and sense of coherence.

**Results:**

The content analysis of the students’ interviews revealed three main categories before the assignment and four main categories subsequently, displaying a broad variety of perspectives. Quantitative analysis identified that 3.2% of the students showed moderate secondary traumatization, and a correlation emerged between the number of shifts and symptom severity of secondary traumatization. The students displayed significantly reduced scores for depression and anxiety, when compared to a sample of first-year medical students. Sense of coherence was identified as a protective factor concerning secondary traumatization.

**Conclusion:**

A rather small proportion of the medical students working in the reception center displayed explicit symptoms of psychological strain in terms of secondary traumatic stress. Due to their assignments, students were able to improve their cultural awareness, which they reported to be highly relevant for their future occupation. In view of increasing globalization, theoretical and practical courses on issues of flight and global health might therefore be implemented as an obligatory part of medical curricula.

**Electronic supplementary material:**

The online version of this article (10.1186/s12909-019-1730-8) contains supplementary material, which is available to authorized users.

## Background

In parallel with the global increase in armed conflicts, a continuous rise in the number of people fleeing the fighting has been observed over the past years. Reaching levels unprecedented in history, it is estimated that by the middle of 2017, nearly 65.6 million people had been forcibly displaced due to persecution, violence and torture worldwide [1]. In 2016, a total of 363.348 refugees, mostly from African countries and Syria, arrived in Europe via the Mediterranean Sea, according to the International Organization for Migration [2]. These refugees frequently suffer from physical as well as psychological conditions. In this regard, studies have shown high prevalence rates of infectious diseases among newly arrived asylum seekers from several origins [3, 4], mainly with respect to tuberculosis [5] and cases of *Plasmodium vivax* malaria [6]. Furthermore, recent systematic reviews describe prevalence rates of 30.6% for post-traumatic stress disorders (PTSD) and 30.8% for depressive disorders among refugees [7, 8], presumably as a reaction to the experience of violence and displacement in their home countries, to flight, or to postmigration stressors, e.g. at the communal shelters. Therefore, in light of the progressing globalization of culture, trade, disease and crises, global health issues are becoming increasingly relevant for the doctors of tomorrow [9, 10].

The 2010 worldwide survey on global health education in medical undergraduate curricula emphasized the gradual development of global health teaching [11]. However, global health issues are still far from being considered as a core part of medical curricula [12], and initiatives specifically focusing on refugee health care provision are even less frequent. Programs range from simulation-based medical education (SBME) scenarios, including infectious disease management in a refugee camp [13], to refugee-patient encounters aiming to enhance cultural awareness and sensitivity [14], elective refugee health curricula [15] and primary care curricular programs [16]. Such programs comprise clinical sessions with refugee patients and educational workshops [17], as well as “refugee health nights” in which refugee patients, students and family physicians get together to discuss refugees’ medical needs and clinical encounters [18]. However, to the best of our knowledge, reports on medical students working voluntarily in refugee reception centers and on the resulting psychological strain after close contact with traumatized refugees are still lacking.

After arriving and being sheltered in a reception center, asylum seekers, potentially suffering from severe psychological traumatization, come into close contact with various professionals, e.g. doctors, social workers and interpreters [19] while being lawfully registered and undergoing medical examination. This constellation can potentially lead to a “transmission” of trauma-related symptoms from a primarily traumatized person to an initially healthy individual, referred to as secondary traumatization [20]. The concept of “secondary traumatic stress” (STS) describes the emergence of post-traumatic stress disorder (PTSD) symptoms within primarily non-traumatized persons due to close contact with traumatized individuals [21, 22]. This notion is extended by the concept of “vicarious traumatization” (VT), which includes not only the emergence of emotional and PTSD-like responses, but also changes in the affected individual’s view of the world, self-capacities and psychological beliefs [20, 23]. STS and especially VT are thought to have widespread economic effects [24]. Consequently, to prevent psychological impairment in terms of STS or VT in health care personnel working with traumatized refugees, the early identification of psychological change and/or symptoms is paramount. Based on investigations with PTSD-Patients, it can be assumed that identifying signs of STS or VT at an early stage and promptly giving therapeutic support can prevent symptoms to increase and will therefore result in a better outcome [25].

As yet, there is a lack of insight as to how medical students evaluate their learning success when working in a refugee reception center, and whether they experience psychological strain in terms of secondary traumatization. This is of particular importance: On the one hand, the working environment in a reception center gives students the opportunity to gain knowledge about a wide range of different diseases, including psychological disorders and rare tropical infections [4, 7]. On the other hand, working in a setting which involves close contact to traumatized individuals can result in psychological distress for the students [26–29]. Such distress may decrease learning achievements [30] and could even become chronic, leading to considerable limitations in mental well-being [22].

In a previous study conducted in 2016, Bugaj et al. investigated psychological stress and possible protective factors in a sample of *n* = 293 first-year medical students. It was shown that students with a secure attachment style experienced significantly less stress than students with insecure attachment styles [31]. In addition, a positive “Model of the Self”, as part of the dimensional analysis of adult attachment, was also identified as stress-protective in medical students [31]. The above mentioned study by Bugaj et al. examined the general experience of stress in medical students. However, it can be assumed that additional context-specific stressors come in to effect during the voluntary assignment of medical students in a refugee reception center. In order to better understand the experiences, learning achievements and stressors of this specific working context, we conducted a prospective study using a mixed-methods approach. Herein, we investigated how medical students experienced their work in an outpatient clinic for refugees, situated in a reception center. In detail, the study aimed to investigate (1a) medical students’ motives for volunteering to work in a reception center and their experiences in this working environment, and (1b) the students’ learning progress while working in the reception center. We investigated these aspects by conducting semi-structured pre-post interviews with a subgroup. Further, we used psychometric questionnaires to explore (2a) the resulting psychological strain, and (2b) possible protective factors.

## Methods

### Study design

In the present study, we applied a mixed-methods approach following a concurrent triangulated design in accordance with the classification of mixed-method designs by Creswell and Clarke [32, 33]. Thus, we concurrently undertook quantitative and qualitative assessments. Our prospective study comprised qualitative interviews with a pre-post design and psychometric questionnaires for a cross-sectional quantitative descriptive approach. According to the recommendations of Creswell et al. (2003), results were equally weighted and examined for possible convergence [33]. By administering a mixed-methods approach, we aimed to generate a diverse and multifaceted perspective on this new learning environment for medical students. Our goal was to examine both the potentially resulting psychological burden and the subjective impressions of the students working with refugees. For this purpose, all 89 medical students of the University of Heidelberg, Germany who volunteered to work in the outpatient clinic of the Medical Treatment Center of the Heidelberg-Kirchheim Refugee Reception Center “Patrick Henry Village” (PHV) [34] to support the early implementation phase were invited to participate in the study. The various aspects of the present investigation are reported in line with the “Good Reporting of A Mixed Methods Study”(GRAMMS)-Criteria [35].

### Student sample

All volunteer medical students working at the outpatient clinic in PHV between January and June 2016, who agreed to participate, were included in the study. Participant recruitment for the pre-interviews took place before students began working in the reception center between January and February 2016. All the participants who had agreed to a pre-interview were interviewed before their first shift. The cross-sectional questionnaire study was conducted between March and June 2016. Post-interviews were held in June 2016. The inclusion criterion for the post-interviews was a minimum of five assignments in the outpatient clinic of PHV.

### The refugee reception center Patrick Henry Village in Heidelberg-Kirchheim

Germany is an important destination for many refugees arriving in Europe, as reflected by the increasing number of asylum applicants at the Federal Office for Migration and Refugees (Federal Ministry for Migration and Refugees, BAMF). When applying for asylum in PHV, newly arrived refugees have to undergo several processes, including registration and medical examination [36]. A medical outpatient clinic was set up in the former dental clinic of PHV [34] and is run by registered physicians in Heidelberg and physicians from the University Hospital in Heidelberg. Refugees can receive medical care in general medicine, pediatrics, gynecology, tropical medicine, and psychosocial medicine [37].

### Role of medical students in setting up the PHV medical center and medical students’ tasks and learning goals

From the very beginning, medical student body representatives were an integral part of the PHV medical center’s steering committee. The committee was founded in November 2015 under the auspices of the Public Health Department, Heidelberg, in order to develop, implement, and maintain the refugee medical center within the PHV reception center [34], encompassing the above-mentioned outpatient clinics. The steering committee additionally comprises Public Health Department and regional council representatives, the refugee camp operator European Homecare (EHC), registered medical community representatives (general practitioners, gynecologists and pediatricians), administration representatives of the University Hospital Heidelberg, university medical representatives from the fields of general medicine, pediatrics, gynecology, tropical medicine and psychosocial medicine (psychiatry, psychosomatics and medical psychology), as well as employees of the Institute for Public Health of the University of Heidelberg. Medical students volunteered to work to develop, assist and accompany the implementation of the refugee camp’s outpatient clinic. Their tasks in the outpatient clinic were defined on a structural, interactional and medical level, reflected in pre-established learning goals (see Table 1). As recommended by Edwards and colleagues [38], an introductory course and supervision were provided.

**Table 1:** Learning goals on the structural, medical and interactional level

On the structural level	
- Dealing with computer systems	
- Importance and management of patient documents / files	
- Laboratory requests	
- Printing / filling out / forwarding (Fax) prescriptions	
- Accounting	
- Overview of expenditures, legal status of medicines (prescription or non-prescription), costs of medication	
- Integration of the medically indicated measures into the patient’s everyday life	
- Interaction with various health system bodies (pharmacies, laboratories, medical specialists, offices etc.)	
- Transfers	
- Documentation	
On the medical level:	
- Blood sampling	
- Dressing / wound care	
- Inspection	
- ECG	
- Blood pressure, pulse, temperature	
- Communication	
- Documentation, communication	
- Assistance during sonography	
- Medication plans / prescriptions	
On the interactional level:	
- Overcoming language barriers (among other things, encouraging and motivating potential translators, etc.)	
- Identification of cultural peculiarities / differences	
- Respect for and dealing with cultural differences	
- Creating a basis of trust that gives patients the feeling of being cared for	
- Ensuring that important information is not only understood, but also accessible in the long term	
- Identification of orientation markers (temporal / structural / organizational etc.)	
- Dealing with anxious patients against the background of difficult organizational situations (often short-term transit, uncertain future etc.)	
- Dealing with physically and emotionally highly stressed patients / employees	
- Forwarding information to the right places	
- Networking with different health system bodies / telephone contacts	

### Structural aspects of the assignment of the medical students at PHV

Before the students started to work at PHV, they attended an approximately two-hour introductory course on the premises of the University Hospital Heidelberg. The students were given the following information: a) the premises of PHV, b) organizational procedures, c) aspects of flight and forced migration, d) the asylum procedure in Germany, e) frequent physical disorders of refugees, f) mental disorders of refugees, g) intercultural communication. In addition, all participants received a manual which included detailed instructions on how to use the computer software and listed important contact details. The students entered their names in a digital shift plan according to their wishes and availability. The shifts at PHV were organized from Monday to Friday. Every day, there were three consecutive shifts of 3.25 h each. The first shift began at 8.30 am and the last shift ended at 6 pm. Two to three students were assigned to an individual shift. The students’ tasks mainly included the admission and forwarding of patients; depending on the capacities, the students also assisted in the doctors’ examination rooms and carried out a basic anamnesis or different diagnostic procedures. The students were then given the opportunity to make their own diagnosis (see Table 1). In order to facilitate communication, students were asked to enter unfinished tasks, new or special features into an online logbook.

### Ethics

Ethical approval was granted by the ethics committee of the University of Heidelberg (Nr. S-694/2015). Study participation was voluntary and all candidates were assured of anonymity and confidentiality. The study was conducted in accordance with the Declaration of Helsinki (most recent version: Fortaleza, Brazil, 2013). Written consent was obtained from all participants.

### Sociodemographic data of student sample

At the beginning of the interviews, as well as prior to the questionnaire-based cross-sectional study following their assignments, participants completed questionnaires regarding sociodemographic information and previous work experience (see Additional file 1: Table S1). Group characteristics, such as age (years), gender (female/male), previous clinical internships, previous course of study or education, experiences of volunteer work and the desired medical specialty, as well as the respective motivations and the need for psychosocial support of the participants were assessed.

### Qualitative assessment: semi-standardized pre-post interviews

The pre- and post-interview questions were developed based on an in-depth literature review as well as expert discussion (*N* = 4, all of whom were experienced in qualitative research). The interview manual was constructed in a semi-standardized manner [39–42] and contained leading open-ended questions with a focus on motivations, expectations, experiences and reflections regarding the students’ assignment in PHV, followed by encouraging questions if required (see Additional file 1: Table S2). In line with the “Consolidated Criteria for Reporting Qualitative Research” (COREQ)-checklist [43], we informed all participants about the study’s background, objectives and procedure. A trained female research assistant (MPJ) conducted individual face-to-face interviews or interviews via phone, following the semi-standardized interview manual. The face-to-face interviews were conducted on the premises of the University of Heidelberg or at PHV. On average, the pre-interviews took 7 min and the post-interviews lasted for 20 min. Dialogues were audiotaped. The interviewer (MPJ) was supervised by an experienced colleague (CN). Participants received textbooks or coupons for specialist bookstores (value 10 €) as compensation for their participation.

### Qualitative content analysis

After verbatim transcription of the 16 pre- and 13 post-interview audio files, a qualitative content analysis was performed following the principles of inductive category development [44]. First, we conducted an open coding of all of the 29 interview transcriptions line by line. In detail, single or few sentences were identified as a code, representing the most elemental unit of meaning [45]. Next, the codes were summarized into relevant themes for each participant, using the software “MAXQDA” [46]. As themes were recurrent among different participants, they were compared and adapted until a number of relevant themes for all participants could be defined. Two independent analyzers assigned the respective codes to specific themes and subsequently discussed them to reach consensus (MPJ, CS; investigator triangulation). In the final step, themes were consolidated into relevant categories.

### Quantitative assessment: evaluation of experiences in PHV

To evaluate the students’ experiences more specifically, a nine-item questionnaire was developed based on an in-depth literature review. The nine statements included in the questionnaire focused on organization, expectations, experiences and learning progress of the students in the reception center and could be answered on a 7-point Likert scale from completely disagree to completely agree (see Additional file 1: Table S3). Mean values and standard deviations were identified.

### Quantitative assessment: evaluation of psychological strain

We assessed secondary traumatization using the German Questionnaire for Secondary Traumatization (FST = Fragebogen für Sekundäre Traumatisierung) [21, 47]. The questionnaire includes 31 items covering the four PTSD symptom clusters according to the DSM-V and additionally assessing sense of threat and safety behavior. Internal consistency of the FST total score is high with Cronbach’s α = 0.94 [47]. In the authors’ sample, participants scoring between 65 and 82 points were classified as experiencing moderate secondary traumatization, while participants scoring above 82 were classified as suffering from severe secondary traumatization [47]. In the present study, students were asked to rate how often the 31 symptoms occurred during the worst week of their assignment on a 5-point Likert scale (1 = *never* to 5 = *very often*).

Depression was assessed using the German version of the PHQ-9 depression module [48] of the Patient Health Questionnaire PHQ; [49]. The PHQ-9 depression module is designed to assess depressive symptoms, disorder severity and symptom development according to the DSM-V [50] and shows very good validity measures [51, 52]. The PHQ-9 has high internal consistency with Cronbach’s α = 0.89 [53]. The nine items assess the occurrence of depressive symptoms in the past 2 weeks, with a sum score ranging from 0 (no depressive symptoms) to 27 (all symptoms occurring daily). The recommended cut-off to distinguish between clinical and non-clinical populations is 10 or above.

Anxiety was assessed using the Generalized Anxiety Disorder Seven-Item Scale [GAD-7; 54], which assesses the presence of anxiety symptoms over the past 2 weeks in line with DSM-IV criteria. Internal consistency is high (Cronbach’s α = 0.89) [54]. Items are scored on a scale from 0 (*not at all*) to 3 (*nearly every day*). A score of 10 or higher is interpreted as indicative of significant anxiety, and scores above 15 indicate severe anxiety [55, 56].

To assess health-related quality of life, we administered the SF-12 [57], which is an economical short form of the internationally established SF-36 Health Survey [58, 59]. The SF-12 comprises 12 items selected from the SF-36 based on their relative efficiency or psychometric performance across eight dimensions of health (e.g. pain, vitality, psychological functioning). The SF-12 is scored to yield two summary scores, for physical and mental health. The SF-12 displays high internal consistency in the two subscales concerning mental and physical wellbeing [60]. A large German norm sample exists for the SF-36 [61], which can be used for the interpretation of the SF-12 [62].

Attachment style was assessed using the German version of the original Relationship Questionnaire RQ-2, [63], German version, Asendorpf, Banse, Wilpers, & Neyer 1997, [64]. The RQ-2 is a four-item questionnaire designed to measure adult attachment style, with each item describing one of the four established attachment styles (secure, preoccupied, fearful-avoidant, dismissive-avoidant). The items are rated on a 7-point Likert scale and can be analyzed dimensionally with scores for each of the four possible categories and categorically by reporting a *model of self* and a *model of others* in close relationships.

The German version [65] of the Sense of Coherence Scale by A Antonovsky [66] was employed to assess sense of coherence (SOC), an attribute of Antonovsky’s concept of salutogenesis [67]. The SOC Scale is a 29-item, multi-dimensional scale that assesses how people view life and, in stressful situations, identify, use and reuse their general resistance resources to maintain and develop their health. Its three main dimensions, comprehensibility (four items), manageability (five items) and meaningfulness (four items), are scored on a 7-point Likert scale with two anchoring responses, “never” and “very often”. A large community-based sample (*n* = 1944) with age- and gender-specific standard scores exists for the German population [68, 69].

### Quantitative statistical analysis

Data are presented as means ± standard deviation (SD) or medians. For the sample description, descriptive statistics were computed. Normally distributed data were analyzed using parametric tests; otherwise, non-parametric tests were employed. A *p*-value < 0.05 was considered to be statistically significant. The software package IBM® SPSS® Statistics Version 22 was used for statistical analysis. Since the above mentioned study by Bugaj et al. (2016) [31] examined the general stress load of medical students, we used the results of the PHQ-9, GAD-7, SF-12 and RQ-2 questionnaires as a “baseline”-measurement. In regard to the assessment of the Sense of Coherence, we used the psychometric results of the SOC scale in a large representative norm sample as the “baseline”-measurement, which were taken from a study by Hannöver et al. (2004) [69]. By comparing the psychometric results of the present study with the results of the studies conducted by Bugaj et al. and Hannöver et al., we aimed to estimate the extent of context-specific stressors in a reception center for refugees on the psychological burden of medical students. The group comparisons were carried out using Student’s t-test for independent samples. Correlations were calculated using Pearson’s correlation coefficients.

## Results

### Sample description and response rate

**Fig. 1:**
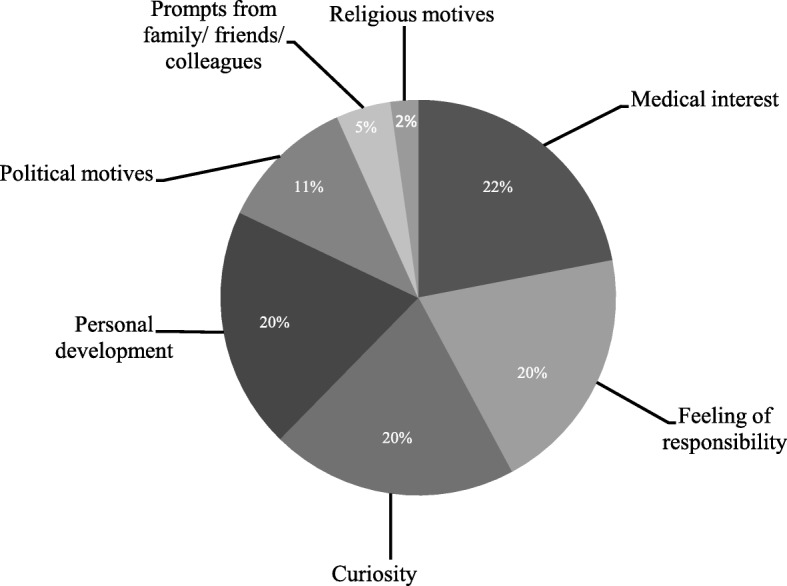
Motivations for volunteering in the reception center The pie chart depicts the various proportions of main motivations of the *n* = 62 medical students.

The sociodemographic characteristics of the sample are displayed in Table 2. From a total of 89 medical students, *n* = 62 students participated in the investigation, corresponding to a response rate of 69.6%. The main reasons given by students for not participating in the study were 1) lack of time or 2) not wanting to reveal personal information. Interviews were held with a subsample of *n* = 16 students for the pre interviews and *n* = 13 for the post interviews. The students showed a high diversity of motivations to volunteer in the refugee reception center in parallel to their core curriculum; these are depicted in Fig. 1. 8.1% of the students deemed that psychological support, i.e. supervision, was necessary following the assignments. Students’ wish for psychosocial support was not correlated with gender or the number of shifts in the reception center. At the beginning of the investigation, some of the participants reported that they were currently undergoing psychotherapeutic or psychopharmacological treatment (see Table 2).

**Table 2:** Sociodemographic characteristics of the assessed medical students

Psychosocial assessment (*n* = 62)	
variable	mean (SD)
Age [years]	23.63 (2.40)
Gender [female / male]	79% / 21%
Year of study [years]	3.35 (1.31)
Number of shifts	4.08 (3.26)
Current treatment (having started prior to assignment)	
-Psychotherapeutic	1.6%
-Psychopharmacological	1.6%

### Qualitative assessment: results of pre-post interviews

With regard to the qualitative analysis of the interview transcripts, 210 single codes from the pre-interviews and 367 single codes from the post-interviews were identified. From these codes, three main categories were derived regarding the pre-interviews and four main categories regarding the post-interviews. The main categories of the pre-interviews were (A) prior experiences with refugees or flight, (B) motivations for voluntary assignment and (C) expectations prior to the assignment. Each of the main categories (A to C) contained 2–7 themes, which were further differentiated into subthemes. The main categories of the post-interviews were (D) subjective experience during the assignment, (E) subjective experiences after the assignment, (F) reappraisal and (G) meaningfulness. Each of these main categories contained 2–5 themes, which were further differentiated into subthemes. Citations for each definition are depicted in Table 3.

**Table 3:** Quotations from semi-structured interviews with subgroup of students

Quotations from pre-interviews	
Category A: *Prior experiences with refugees or flight* (36 codes)	
• *“In the hospital I already had some encounters with refugees, which I found very positive (…) I found it a little bit difficult that there were certain expectations (from the refugees)” (B)*	
• *“My grandparents arrived as refugees in Germany after the Second World War” (H)*	
• *“If there’s something in the news, once again everybody talks about it. It is an issue which divides (the opinions of) everybody. I mean, it is okay now; we are here at the university in Heidelberg and here we have a (common) opinion. But what about other communities? (…) I do try not to discuss too much, because you know, there won’t be an easy solution. I don’t think this is the aim of the Patrick Henry Village. You don’t try to solve the problem, but somehow try to give aid to those, who are there, who need medical help.” (C)*	
Category B: *Motivations for voluntary assignment* (55 codes)	
• *“(I want to get) a more realistic perspective on the whole refugee issue; and perhaps I can learn from it some modesty and gratitude for the life conditions in which I grew up and in which I live now.” (B)*	
• *“I think everybody can contribute a little bit (…) and then certain things may be realized, which would not have been possible otherwise.”(G)*	
• *“I (consider) us, as a community, as well as me, as an individual, to have a duty (to give aid)” (M).*	
Category C: *Expectations prior to the assignment* (119 codes)	
• *“In fact, I expect a range of diseases that are not typically taught in medical curriculum. (…) and I also think that (…) a lot of them (refugees) come to Germany with traumatic experiences, so that giving aid is very difficult.” (B)*	
• *“I just don’t know what I’ll be confronted with. I think when there are severe issues, it won’t just simply pass me by.” (G)*	
• *“What about infectious diseases? (…) tuberculosis is very frequent in those countries.” (D)*	
Quotations from post-interviews	
Category D: *Subjective experiences during the assignment* (248 codes)	
• *“(I communicated) with general practitioners, if they had issues; or someone from the university called me with an issue. (It was important) to communicate and solve problems within the team.” (R)*	
• *“And then something happens or a doctor doesn’t make progress (in examining a patient) and everybody has to wait. Consequently the atmosphere (in the waiting room) can rapidly become stressed.” (R)*	
• *“(For example, when) they (refugees) just didn’t understand what kind of pills they had to take. Due to the language barrier it (the issue) was magnified and sometimes it got very loud.” (W)*	
Category E: *Subjective experiences after the assignment* (31 codes)	
• *“(It is) fascinating to switch from the lives in which those people (refugees) are captured, back to my own, normal life. A life (of refugees), in which the most important role is played by where you are and where you can stay and in which other people decide if you are allowed to move along or not. This is quite interesting (…) to deal with it, to listen to what the media and others report. It was quite distressing for me, even dominating my everyday life for a time (…) so I talked and read a lot about it and even dreamed that I was in a war.”(Y)*	
• *“That means I can’t really detach (the experiences in PHV) from what happens later on. But in fact, I felt well mostly. So, I ride my bicycle for half an hour. Then I have some time to focus on the rest or the other world, so to speak. And then it is usually OK again.” (R)*	
• *“I can state, except for the last shift, I really felt well the other shifts. I felt I was efficient, because I accomplished something on this day.” (O)*	
Category F: *Reappraisal* (31codes)	
• *“(I learned) that you can’t just consider them (refugees) overall to be a homogenous mass; instead there are arriving as very, very diverse human beings.” (Y)*	
• *“At the beginning I thought ‘The refugees need help, every one of them is kind and grateful’. Of course, there are such friendly patients among the people. But working in PHV, I recognized that some others are quite unkind. In fact, you have a naive idea in your head that everybody is really happy and everybody is kind. Well, in fact we are kind. However, as I said before, there was that one (refugee) screaming so loud. Sometimes, situations weren’t quite friendly.” (Q)*	
• *“Since then, I consider medicine to be a more political issue. The basic right for medical provision is in fact supreme; it is the supreme human right. I’m so aware of this now.” (Ö)*	
Category G: *Meaningfulness* (57 codes)	
• *“(I had the feeling) that I was doing something, without which it wouldn’t have worked out as well as it did. Till then I had the feeling of affecting something, even if it was marginal. But when I sat in the doctor’s office, looking over his shoulders, like in a usual traineeship and taking blood samples means constraining (of the procedures), then you have to be clear about what you are doing and who’s benefiting from this.” (Z)*	
• *“(I think) that it was and still is worth going there (PHV). That this is a kind of work which I, being intrinsically motivated, enjoy performing and which gives something back to me. And that it is nice, to give aid to people in need.” (Ä)*	
• *“In any case, I learned a lot. Not only on a medical level, but I also became aware of a lot of organizational stuff and about the procedures which you perform in such a medical center.” (S)*	

#### Definitions of main categories and themes of pre-interviews

##### Prior experiences with refugees or flight (36 codes)

Some of the students already had experiences with flight through internships or clinical traineeships. Furthermore, a few participants recalled a history of flight in their own family. The students often debated the issue of refugees and flight with colleagues, friends and family prior to their commitment and were often confronted with concerns from others about their assignment.

##### Motivations for voluntary assignment (55 codes)

Important motivations of the students mainly concerned social and political issues. In the context of political aspects, some participants felt a need to underline their views as a counterbalance to xenophobic or conservative opinions. Moreover, the students hoped to broaden their personal and occupational horizons by encountering people from other cultures. The feeling of satisfaction from being able to give aid to people in need also played an important role.

##### Expectations prior to the assignment (119 codes)

A large proportion of medical students expected and worried about the administrative and organizational tasks that they would have to undertake in the reception center PHV, but was also prepared to perform medical tasks if required. However, they considered the main challenges to lie in the anticipated language barriers and cultural differences. With regard to anticipated *medical* challenges, the students were worried about insufficient medical equipment on site. The participants mentioned infectious and exotic diseases, mainly tuberculosis, as an anticipated challenge. Nevertheless, they also expected high numbers of refugees with severe psychological traumatization. Some of the participants estimated that learning about the refugees’ potentially traumatic background stories or experiencing severe medical conditions of refugees might affect them outside of their assignment, or even lead to psychological strain.

#### Definitions of main categories and themes of post-interviews

##### Subjective experiences during the assignment (248 codes)

Medical students often perceived themselves in the role of a mediator between the different occupational groups in the reception center, e.g. refugees, doctors, social workers, security personnel and interpreters. An important interactional challenge lay in the language barrier with which students were faced. In addition, they reported challenges in the context of cultural differences, especially issues of gender-specific interaction or concerning the performance of physical examination. With respect to medical challenges, participants mentioned the handling and treatment of patients with chronic or infectious diseases as well as psychologically strained or even traumatized individuals, while not having sufficient capacities to offer proper treatment. Through assisting in the doctors’ offices or reading the doctors’ letters, the participants learned in detail about the refugees’ personal background stories and strokes of fate. In this context, a central theme was the refugees’ insufficient access to medical support on their flight, as well as the major consequences of torture and the loss of close relatives through persecution and war. Especially in the latter case, the language barrier was reported to be a major obstacle to building up therapeutic trust. The medical students perceived the intensity, frequency and quality of contacts with the refugees in various ways, evoking a broad range of emotions. In many cases, participants indicated a feeling of helplessness concerning the individual situation in the reception center on the one hand, and the refugees’ background stories, their flight and the necessity for social provision on the other. Positive emotions, especially pleasure and fulfillment, were often evoked due to contact with children or pregnant women.

##### Subjective experiences after the assignment (31 codes)

Following their work in the reception center, students concerned themselves with medical as well as organizational aspects of their experiences, reflecting on the broader perspective of global flight and refugee health issues. Some students reported having been preoccupied with remembering, at times even for weeks, the individual situations in the outpatient clinic or traumatic background stories of the refugees. Subsequently, they talked to friends, family and colleagues in order to cope with their negative experiences of the assignment. However, a major proportion of students did not consider psychological support, e.g. supervision, to be necessary.

##### Reappraisal (31codes)

Some participants reported changing their view on refugees, stating it to now be “more realistic”. Most students underlined that their view on the world had been confirmed rather than changed due to working with refugees. However, they highlighted a newfound awareness of the comfort of their own life and their “privileged” standard of living. From this, they often derived a kind of responsibility and moral duty to help people in need. Finally, they stated that they gained knowledge concerning the political and ethical dimensions of medicine.

##### Meaningfulness (57 codes)

The satisfaction from giving aid and being useful as well as the gain in medical, organizational, social and political knowledge were appraised as being of major personal benefit. In summary, most of the participating students appreciated the opportunity to become involved in the reception center and thus broaden their personal and cultural horizons.

### Medical students’ rating of their experiences in the reception center

The students’ responses to the nine statements of the questionnaire are depicted as mean values and standard deviations in Table 4. Participants reported only a small increase in medical knowledge (M = 2.63; SD = 1.74) but a major gain in knowledge and skills concerning interactional (M = 5.06; SD = 1.38) or organizational issues (M = 5.29; SD = 1.19).

**Table 4:** Evaluation of assignment in the reception center

Medical students’ (*n* = 62) rating of experiences during refugee work		
Item	Mean	SD
The assignment in PHV was well organized	4.29	1.44
I enjoyed the assignment in PHV	5.94	1.18
The number of students per shift was completely appropriate	5.66	1.17
The range of tasks met my expectations precisely	4.26	1.16
I felt well supervised	4.02	1.37
The experiences I had are important for my future occupation	4.40	1.78
I gained knowledge on the medical level	2.63	1.74
I gained knowledge on the organizational level	5.29	1.19
I gained knowledge on the interactional level	5.06	1.38

Medical students’ evaluation of work in the reception center, assessed by nine statements rated on a 7-point Likert scale ranging from 1 = fully disagree to 7 = fully agree. The results are depicted as mean values (Mean) and standard deviations (SD)

### Secondary traumatization and psychological strain in medical students

Quantitative results for psychological strain measures are depicted in Table 5. Regarding individual FST scores, a moderate secondary traumatization due to the work in the reception center was present in 3.2% of the examined students. No students were found to be suffering from severe STS. Moreover, there were no gender differences with regard to the FST scores (*p* = .068). A significant correlation was identified between the number of assignments in PHV and the FST sum score (*r* = .256, *p* = .045). The participants’ sum score for depressive symptoms and anxiety was significantly lower compared to a norm sample of first-year medical students [31]. In summary, 17.7% of the participants showed PHQ-9 scores corresponding to mild depressive symptoms. 11.2% of the students displayed mild anxiety symptoms and 3.2% showed moderate anxiety symptoms. Health-related quality of life measured by the SF-12 indicated significantly higher scores for mental health when compared to a population of first-year medical students [31]. No gender differences were detected.

**Table 5:** Psychological strain and attachment style: Descriptive data and correlations

Psychological assessment– descriptive statistics (*n* = 62)							
instrument	mean (sd)		norm sample		significance		
			mean (sd)	n	t	df	*p*
FST [31–155]	38.52 (7.91)		–	–	–	–	–
PHQ [0–21]	2.22 (2.45)		6.03 (4.19)^a^	290	−6.907	334.917	<.001**
GAD [0–21]	2.46 (2.68)		5.34 (4.24)^a^	290	−5.130	322.314	<.001**
SF-12 [0–100]							
Physical health score	56.06 (3.03)		55.06 (5.08)^a^	290	1.493	332.081	0.136
Mental health score	52.03 (6.52)		45.33 (10.33)^a^	290	4.899	322.881	<.001**
RQ							
Model of self [−12 − + 12]	4.58 (3.34)		3.81 (3.633)^a^	279	1.525	222.417	0.129
Model of other [−12 − + 12]	0.81 (3.57)		1.03 (3.260)^a^	279	−0.477	177.991	0.634
*Secure*	5.16 (1.35)		5.18 (1.389)^a^	280	−0.103	207.636	0.918
*Dismissing*	2.47 (1.79)		3.73 (1.605)^a^	282	−5.479	174.087	<.001**
*Preoccupied*	1.98 (1.09)		2.33 (1.399)^a^	282	−1.849	268.672	0.066
*Fearful*	3.87 (1.67)		2.74 (1.842)^a^	281	4.443	226.633	<.001**
SOC-29 [29–203]	151.42 (16.63)		155.0 (23.0)^b^	4002	−1.227	490.21	0.22
Psychological assessment – correlation analyses (*n* = 62)							
	FST		PHQ		GAD		
	r	p	r	p	r	p	
RQ – model of self	−.181	.159	−.184	.152	−.029	.825	
	r	p	r	p	r	p	
RQ - model of other	−.047	.718	−.264*	.038	.074	.568	
	r	p	r	p	r	p	
SOC-29	−.414**	.001	−.428**	.001	−.487**	<.001	

*FST* Questionnaire for Secondary Traumatization, *PHQ-9* Patient Health Questionnaire depression module, *GAD-7* Generalized Anxiety Disorder Scale, *SF-12* 12-item Short Form Health Survey, *RQ* Relationship Questionnaire, *SOC-29* Sense of Coherence Scale. ^a^ [31], ^b^ [69]

*p < .05; **p < .01

### Attachment style, sense of coherence and their correlation with psychological strain in medical students

The results of the measures for attachment style, sense of coherence and their respective correlations are depicted in Table 5. In terms of attachment style, high scores for “model of others” correlated with low scores for depressive symptoms. Furthermore, high scores for sense of coherence correlated significantly with low psychological strain values in terms of secondary traumatization, depression and anxiety.

## Discussion

The present study is the first to evaluate medical students’ volunteer work in a refugee reception center. The results indicate that a small proportion of the students suffered from psychological strain following their assignment, mainly in terms of secondary traumatization. In this regard, a correlation between secondary traumatization on the one hand and the students’ number of shifts in the reception center as well as a present sense of coherence on the other hand was detected. Subsequently, 8.1% of the participants deemed simultaneous or consecutive psychosocial support to be necessary. During their assignment, the students were confronted with mainly interpersonal and organizational problems as well as medical challenges in terms of infectious diseases or psychological traumatization. Following the assignment, the participants appraised their experiences in the reception center, especially the interactional and cultural experiences, to be relevant for their future occupation as doctors.

In the interviews and in the quantitative rating of their experiences, medical students reported various motivations for volunteering to work in a reception center. “Medical interest” was identified as the main motivation in the quantitative assessment of all medical students (indicated by 22%), followed by “sense of responsibility” (20%) and the “desire for personal development” (20%). In the qualitative in-depth interviews, the motivation of “medical interest” only played a marginal role, with “curiosity” concerning the refugees’ personal background stories and the moral “responsibility” to give aid to these people being the crucial motivators for the subgroup. Irrespective of whether the reason for volunteering in the reception center PHV is more medical or interactional in nature, it is apparent that the future generation of doctors has a major interest in the concerns of and communication with individuals from different cultures and countries [70]. This can be seen against the backdrop of increasing globalization, with its growing influence on areas of everyday life and its expansion into the medical sector due to the recent influx of refugees into industrial countries [9, 13].

Prior to the assignment, the students expected that they would mainly be confronted with organizational and interpersonal challenges, but examining refugees’ vital signs and assisting the outpatient clinic doctors in charge were also part of the students’ expected duties and learning goals. As a further challenge, the participants reported both anticipated language barriers and the handling of physical and psychological conditions of the asylum seekers: They worried about high frequencies of infectious diseases, e.g. tuberculosis, beyond refugees [15, 71], and about encountering individuals with severe psychological traumatization. In this regard, some students even anticipated that they would be psychologically stressed by learning about the traumatic background stories of the asylum seekers. Despite these critical reflections, most of the examined students stated that their assignment was satisfying and generally met their expectations.

After working in PHV, participants reported only a minor gain in medical knowledge, which stands in contrast to their aforementioned motivations prior to the assignment: Instead, they indicated a much greater increase in interpersonal and organizational expertise, which can be referred to as rather “procedural capabilities”. The students frequently saw themselves in the role of a “mediator”, arbitrating between the various professions in PHV and thereby ensuring proper support and organization of medical and social procedures for the refugees in the outpatient clinic. Thus, the students’ area of responsibility was not limited to medical issues, but rather broadly exceeded them, which the participants viewed as a highly valuable experience. Besides the etiology, diagnostics and treatment of physical and mental diseases, the students learned about the organizational and procedural challenges of medical provision for refugees, which are not usually a part of the medical curriculum. A direct link can be discerned between the ascertained high interest in global health issues and the students’ gain in specific “medical-procedural knowledge”. This leads to the important question of whether courses focusing on refugees and global health issues should be *obligatory* in medical curricula, in order to inform every student about global health problems at least to some degree; or whether such courses should be offered as *electives*, as is already the case in various universities around the world [9, 17]. In our view, when discussing this question, the potential psychological strain arising in students working in specific settings involving global health issues needs to be taken into account.

The students experienced their individual encounters with the refugees in different ways and showed various reactions which they articulated in the interviews: A large proportion of the participants reported feeling satisfied by having the opportunity to contribute to the refugees’ well-being and integration. However, some students showed signs of psychological strain after their assignment: They suffered from depressive feelings and intrusive recollections of the refugees’ depictions of traumatic experiences, even for weeks after the assignment, which can be a sign of secondary traumatization (STS) [21]. Additionally, some gained a perspective on their own “privileged” standard of living and changed their views about themselves and the world. The concept of vicarious traumatization (VT) was mainly developed with regard to such changes in cognitive categories after close contact with primarily traumatized individuals, and thus encompasses affective as well as cognitive reactions of initially healthy subjects [20, 24]. These reactions were identified in a minority of the interviewees. Finally, the students concluded that their voluntary assignments in the reception center for refugees were positive, and gave them the chance to broaden their cultural horizons.

Within the framework of the concurrent triangulated design of the mixed-method approach, the results of the content analysis are generally in line with the results of the psychometric questionnaires. The quantitative analysis revealed that 96.7% of the students did not suffer from secondary traumatic stress (STS). The students’ sense of coherence, which did not significantly differ from that of a representative German norm sample, was identified as a protective factor concerning the development of secondary traumatization, depression and anxiety. The protective effect of high values for sense of coherence was already confirmed in previous studies regarding secondary traumatization [72, 73], but also in the context of primary traumatization in terms of PTSD [74]. The participants showed significantly lower values for depression (PHQ-9) and anxiety (GAD-7), a significantly higher score for mental well-being (SF-12), a significantly lower rate of dismissing attachment style, but simultaneously a significantly higher rate of fearful attachment compared to a sample of first-year medical students in a study conducted by Bugaj et al. [31]. These findings may be due to a selection effect, as participation was exclusively voluntary. This notion leads to the assumption that individuals who are already suffering from depressive feelings or interpersonal difficulties, being associated with dysfunctional attachment, are probably unlikely to volunteer in a reception center.

Taken as a whole, the majority of students felt that the assignment in the reception center for refugees broadened their horizons regarding cultural aspects and that they gained a lot of knowledge. Increasingly, the future generation of physicians will be faced with matters of globalization in general, and global health in particular. In this regard, it is important that medical students develop an awareness of cultural differences and particularities, and are sensitive towards these aspects [75]. As people who encounter individuals from diverse cultures in their working environment, doctors and medical students must meet the demands of a progressing “medical globalization”. Apart from being a challenge, these new psychosocial and medical developments also bring the opportunity for medical students to gain knowledge and skills in a working environment that differs from their usual internships. Therefore, we would like to advocate that obligatory theoretical and practical courses will be included in the medical curriculum. In addition, the basic topics from the obligatory classes could be intensified in additional elective seminars. However, the present study showed that some of the medical students are at risk to develop secondary traumatization after working with traumatized refugees. We therefore propose that students attend psychoeducational courses in which they learn about psychological burden and secondary traumatization as potential consequences of their assignment [76] prior to the obligatory courses. These introductory courses were held for the students participating in our study. During their placement in a reception center, individuals should receive frequent supervision by trained psychotherapists [24]. The aim of this support should be to 1) prevent psychological distress or secondary traumatization and 2) be able to identify students who show signs of distress due to their working assignments. Additionally, students should be offered the opportunity to confidentially reach out to psychotherapists in case of psychological strain. The “caseload” of the students working in the reception center should be balanced and time for self-care activities should be taken into account [24]. Additionally, when students attend courses in which they are likely to encounter traumatized individuals, they should be regularly screened for signs of psychological burden.

### Limitations

Several limitations of this study should be mentioned: First, in our study we only examined a relatively small number of participants. Nevertheless, compared to the existing literature, this study is the largest assessment of medical students working in a refugee reception center. Moreover, the high response rate to questionnaires in the current study is not reported in the majority of comparable studies, leading to an acceptable comparability of our findings. Second, psychometric data was only collected after the students had finished their assignments in the reception center. In order to evaluate the actual impact this working environment has on symptoms of depression, anxiety disorders and overall well-being, students would have to be assessed before they start their assignment. However, this restriction does not apply to the measurement of secondary traumatization because the questionnaire (FST) specifically refers to a defined situation in the past. In the case of our study, this situation was the assignment in the reception center. Although the interviews were conducted only in a subsample of students, the codes and categories resulting from the qualitative content analysis gradually reached proper saturation. Third, the above-mentioned selection effect arising from the fact that students’ work in the reception center was voluntary might restrict the generalizability of our findings. The cross-sectional nature of the design only allowed us to identify correlations between personality factors and the severity of psychological strain; the causal role of these factors needs to be conclusively confirmed in predictive studies.

## Conclusion

The present study indicates that medical students working voluntarily in a reception center for refugees only suffer from minor psychological strain. Nevertheless, individual students displayed explicit symptoms of secondary traumatic stress (STS) following their assignments. Due to their work in the reception center, students were able to improve their cultural awareness, which they reported to be relevant for their future occupation. In view of increasing globalization, theoretical and practical courses on issues of flight and global health should be implemented as an obligatory part of medical curricula, accompanied by the optional provision of psychosocial support.

## Additional file

Additional file 1: **Table S1.** Questionnaire on the sociodemographic data of the medical students. **Table S2.** Manual for semi-standardized interviews with the medical students. **Table S3.** Nine-item questionnaire to evaluate the medical students’ experiences in PHV. (DOCX 29 kb)

## Data Availability

The datasets used and analyzed during the current study are available from the corresponding author on reasonable request.

## Abbreviations

BAMF: Bundesamt für Migration und Flüchtlinge (Federal Ministry for Migration and Refugees)
COREQ: Consolidated Criteria for Reporting Qualitative Research
DSM-5: Diagnostic and Statistical Manual of Mental Disorders-5
DSM-IV: Diagnostic and Statistical Manual of Mental Disorders-IV
EHC: European Homecare
FST: Fragebogen für Sekundäre Traumatisierung (Questionnaire for Secondary Traumatization)
GAD-7: Generalized Anxiety Disorder Seven-Item Scale
M: Mean value
PHQ-9: Patient Health Questionnaire, Depression Module
PHV: Patrick Henry Village
PTSD: Posttraumatic Stress Disorders
RQ-2: Relationship Questionnaire
SBME: Simulation-Based Medical Education
SD: Standard Deviation
SF-12: Short Form (of the health-related quality of life questionnaire)
SOC: Sense of Coherence
STS: Secondary Traumatic Stress
UNHCR: United Nations High Commissioner for Refugees
VT: Vicarious Traumatization
