# Supplementary material for: Motives, experiences and psychological strain in medical students engaged in refugee care in a reception center– a mixed-methods approach
Source: BMC Med Educ. 2019 Aug 5;19:302. doi: 10.1186/s12909-019-1730-8 (PMC6683371; doi:10.1186/s12909-019-1730-8)
Supplement: Supplementary file 1 — Table S1. Questionnaire on the sociodemographic data of the medical students. Table S2. Manual for semi-standardized interviews with the medical students. Table S3. Nine-item questionnaire to evaluate the medical students’ experiences in PHV. (DOCX 29 kb) [file 12909_2019_1730_MOESM1_ESM.docx]

**Appendix**

**Table S1:** Questionnaire on the sociodemographic data of the medical students.

**- Sociodemographic Data -**

Please answer the following questions **before your first shift**.

| **Age:** |  | **Gender:** | |  |
| --- | --- | --- | --- | --- |
| **Family status:** |  | **Years of Study:** | |  |
| **Nationality:** |  |  | | |
| **Civilian service** (if yes, where?): |  | | | |
| Which clinical internships have you already completed? (Please list the medical fields.) |  | | | |
| Have you already completed or started with another course of study / training? | | |  | |
| In which medical field would you like to specialize? | | |  | |
| Have you participated in volunteer work? (Please list the areas in which you have been active.) | | |  | |
| Are you engaged in private / voluntary / professional activities in which you come into contact with **psychosocial problems**? | | |  | |
| **Leisure activities, hobbies:** |  | | | |

| Are you currently in psychotherapy?  If so, since when? | Yes |  | No |  |
| --- | --- | --- | --- | --- |
| Have you ever had any psychotherapy or similar treatment? |  | | | |
| Do you take prescribed medication? (Please list the names.) |  | | | |
| How much alcohol do you drink per week? What kind of alcohol do you drink? |  | | | |
| Do you smoke? How many cigarettes do you smoke per day/week? |  | | | |
| Do you use illegal drugs? What kind of drugs do you use and how often? |  | | | |

| What motivated you to volunteer for an assignment in PHV? Multiple answers possible. | | | |
| --- | --- | --- | --- |
| Medical interest |  | Feeling of responsibility |  |
| Religious motives |  | Curiosity |  |
| Personal development |  | Political motives |  |
| Stimulation through family/ friends/ colleagues |  | Others: |  |

Please answer the following questions **after your last shift**.

| How many shifts did you have in PHV? |  |
| --- | --- |

| Would you advocate psychological support for the assignment? | Yes |  | No |  |
| --- | --- | --- | --- | --- |

**Table S2** Manual for semi-standardized interviews with the medical students.

**- Manual for semi-standardized interviews-**

**Pre-Assessment**

| **Main Open Question 1** | **Encouraging Questions** |
| --- | --- |
| *First of all, I would like to know why you volunteered for an assignment at Patrick Henry Village?* | - *Were there inner motivations? If so, what were the motivations?* - *Did you have external reasons? If so, what were your reasons?* - *Were there political motives?* - *Did you discuss those reasons with colleagues, family, or friends?* |

| **Main Open Question 2** | **Encouraging Questions** |
| --- | --- |
| *How do you imagine Patrick Henry Village?* | - *What are you looking forward to?* - *What makes you curious?* - *What challenges do you expect on an organizational level?* - *What challenges do you expect regarding interactions with the refugees?* - *How do you imagine your contact with the refugees will be like?* - *What challenges do you expect to encounter regarding medical aspects?* - *What fears or worries do you have about your first shift in PHV?* |

| **Main Open Question 3** | **Encouraging Questions** |
| --- | --- |
| *How do you expect the work at PHV to affect you personally?* | - *What kind of effects could it have on your psyche?* - *What kind of effects could it have on your personal attitudes / values?* - *What kind of positive effects do you expect?* - *What kind of negative effects do you expect?* |

**Post-Assessment**

| **Main Open Question 4** | **Encouraging Questions** |
| --- | --- |
| *What kind of experiences did you have during your time at PHV?* | - *Have your impressions changed over time?* - *How did you personally experience Patrick Henry Village?*    - *What was the atmosphere like?*   - *What associations did you have?*   - *How did you experience your contact to refugees?*   - *What feelings did you have about the refugees?*   - *Were there difficult situations?*   - *Were there good moments?* |

| **Main Open Question 5** | **Encouraging Questions** |
| --- | --- |
| *What kind of* ***clinical*** *experiences did you have during your assignment?* | - *Was the contact with the refugees different from contact with patients in a more “normal” context?* - *Did you experience your work as meaningful?* |

| **Main Open Question 6** | **Encouraging Questions** |
| --- | --- |
| *Are there any kind of thoughts or images that are on your mind since you have completed your assignment in the reception center?* | - *Which thoughts or memories are most present?* - *How did you deal with your experiences at PHV?* - *Do you feel burdened by the experiences you had?* |

| **Main Open Question 7** | **Encouraging Questions** |
| --- | --- |
| *There is a phenomenon that people who are in close contact with traumatized patients can also be psychologically burdened. Would you be able to say the same about yourself?* | - *Did you involuntarily remember certain situations over and over again?* - *Have you had bad/unpleasant dreams in connection with your assignment?* - *Have you had sleep disorders?* - *Did/do you feel numb or dull?* - *Did/do you feel listless?* - *Did/do you experience anhedonia?* - *Have you avoided situations which you relate to your activities at PHV?* - *Did/do you feel increasingly alarmed in certain situations?* - *Did/do you feel depressive?* |

| **Main Open Question 8** | **Encouraging Questions** |
| --- | --- |
| *Do you think differently about refugees after your assignment in the PHV?* | - *Have your own values changed since your assignment?* |

| **Main Open Question 9** | **Encouraging Questions** |
| --- | --- |
| *What kind of psychological preparation or psychological support before, during, or after an assignment at Patrick-Henry-Village would be supportive?* | - *What types of intervention would be supportive in your opinion?* - *At which point in time would a psychological intervention be helpful?* |

| **Main Open Question 10** | **Encouraging Questions** |
| --- | --- |
| *Under what conditions would you consider volunteering for another assignment at PHV?* | - *Would you like to change something? If so, what would you like to change?* |

| **Main Open Question 11** | **Encouraging Questions** |
| --- | --- |
| *Is there a general conclusion that could you draw from your experience at Patrick Henry Village?* |  |

**Table S3** Nine-item questionnaire to evaluate the medical students' experiences in PHV”.

**-Evaluation of experiences in PHV-**

Please rate the following statements on a scale ranging from 1=fully disagree to 7=fully agree:

| **The assignment in PHV was well organized** | | | | | | |
| --- | --- | --- | --- | --- | --- | --- |
| **1** | **2** | **3** | **4** | **5** | **6** | **7** |

| **I enjoyed the assignment in PHV** | | | | | | |
| --- | --- | --- | --- | --- | --- | --- |
| **1** | **2** | **3** | **4** | **5** | **6** | **7** |

| **The number of students per shift was completely appropriate** | | | | | | |
| --- | --- | --- | --- | --- | --- | --- |
| **1** | **2** | **3** | **4** | **5** | **6** | **7** |

| **The range of tasks met my expectations precisely** | | | | | | |
| --- | --- | --- | --- | --- | --- | --- |
| **1** | **2** | **3** | **4** | **5** | **6** | **7** |

| **I felt well supervised** | | | | | | |
| --- | --- | --- | --- | --- | --- | --- |
| **1** | **2** | **3** | **4** | **5** | **6** | **7** |

| **The experiences I had are important for my future occupation** | | | | | | |
| --- | --- | --- | --- | --- | --- | --- |
| **1** | **2** | **3** | **4** | **5** | **6** | **7** |

| **I gained knowledge on the medical level** | | | | | | |
| --- | --- | --- | --- | --- | --- | --- |
| **1** | **2** | **3** | **4** | **5** | **6** | **7** |

| **I gained knowledge on the organizational level** | | | | | | |
| --- | --- | --- | --- | --- | --- | --- |
| **1** | **2** | **3** | **4** | **5** | **6** | **7** |

| **I gained knowledge on the interactional level** | | | | | | |
| --- | --- | --- | --- | --- | --- | --- |
| **1** | **2** | **3** | **4** | **5** | **6** | **7** |
